# A Binarized Segmented ResNet Based on Edge Computing for Re-Identification

**DOI:** 10.3390/s20236902

**Published:** 2020-12-03

**Authors:** Yanming Chen, Tianbo Yang, Chao Li, Yiwen Zhang

**Affiliations:** 1 School of Computer Science, Anhui University, Hefei 230601, China; cym@ahu.edu.cn (Y.C.); haseka@stu.ahu.edu.cn (T.Y.); 2 Institute of Computing Technology, Chinese Academy of Sciences, Beijing 100190, China; lichao@ict.ac.cn

**Keywords:** binary neural network, cloud computing, edge computing, person Re-Identification, end devices

## Abstract

With the advent of the Internet of Everything, more and more devices are connected to the Internet every year. In major cities, in order to maintain normal social order, the demand for deployed cameras is also increasing. In terms of public safety, person Re-Identification (ReID) can play a big role. However, the current methods of ReID are to transfer the collected pedestrian images to the cloud for processing, which will bring huge communication costs. In order to solve this problem, we combine the recently emerging edge computing and use the edge to combine the end devices and the cloud to implement our proposed binarized segmented ResNet. Our method is mainly to divide a complete ResNet into three parts, corresponding to the end devices, the edge, and the cloud. After joint training, the corresponding segmented sub-network is deployed to the corresponding side, and inference is performed to realize ReID. In our experiments, we compared some traditional ReID methods in terms of accuracy and communication overhead. It can be found that our method can greatly reduce the communication cost on the basis of basically not reducing the recognition accuracy of ReID. In general, the communication cost can be reduced by four to eight times.

## 1. Introduction

Recently, with the rapid development of the Internet of Things, smart cities is becoming a hot research topic. There are more and more end devices in many cities, which facilitates many research efforts on public safety [1,2] based on video surveillance. As an emerging computing model, edge computing [3,4] plays an irreplaceable role in smart cities, having less communication overhead than the cloud-based method. With the help of edge servers, many tasks can be completed directly on the end devices or edge servers without having to be transferred to the cloud, which greatly reduces the overhead of communication. Some target detection and recognition methods can be deployed on the corresponding devices to find specific targets, such as face recognition [5], behavior recognition [6], ReID [7], and license plate recognition [8]. Among them, license plate recognition is used for deployment in public vehicle safety. Pedestrian recognition, whether it is face recognition or person ReID, has played a major role in maintaining social order and security. For face recognition, it is difficult for ordinary cameras in cities to capture clear faces. By contrast, the ReID approach is easier to implement, and it can be carried out by features such as the clothing, color, and texture of pedestrians.

With the rapid development of deep learning, the current algorithm based on CNN has a much higher recognition accuracy than the traditional algorithm of extracting features manually. The CNN network is constantly changing. Initially, in 2012, Hinto and his student Alex Krizhevsky proposed AlexNet [9]. After that, in 2014, VGG [10] was proposed by the Oxford Visual Geometry Group of Oxford University. At the same time, Christian Szegedy proposed GoogLeNet [11]. Finally, in 2015, Kaiming He et al. proposed the ResNet [12] series of networks (18-ResNet, 34-ResNet, 50-ResNet, 101-ResNet, and 152-ResNet).

There are many algorithms [13,14,15] that use the above CNN network to solve the pedestrian recognition problem. However, these approaches are all cloud based, which transmit the pedestrian images collected from the end devices to the corresponding cloud. This method can deploy CNN models and conduct training and inference in the cloud. Although the pedestrian recognition method deployed on the cloud platform with sufficient computing resources (GPU, etc.) can achieve the highest recognition accuracy, it also brings some problems. For example, when the pedestrian images collected from the end devices or the edge are transmitted to the cloud, this will bring great problems to communication, such as delay and privacy issues due to the bandwidth fluctuation in the transmission process.

Recently, with the rise of edge computing, the neural network model can be deployed on the edge to solve some problems, such as privacy preservation [16] and the above pedestrian recognition problem. This approach solves a number of problems associated with long-distance data transmission, but it also creates new problems. For example, because most common edge devices have limited computing resources, it is hard to deploy a deeper neural network model on the edge, which leads to a lower accuracy of pedestrian identification. In the face of such problems, in this paper, we propose a binarized segmented ResNet model based on the three ends. The ResNet structure will sink to the edge, or even to the end devices. Compared to the mobile-oriented model, the ResNet model can achieve a high recognition accuracy in ReID. Although ResNet is heavier, we deploy a shadow sub-network of ResNet on the end devices, which will greatly reduce the communication cost. In this paper, we take ReID as an application scenario for pedestrian recognition. In the field of ReID, the mainstream methods are still based on the cloud to achieve high recognition accuracy. Our work pays more attention to the application of ReID in actual scenarios, where real time is more important. For example, in a criminal incident, various cameras in the city can be used to compare a series of pedestrians through the deployed ReID model to find the criminal at some point.

The contributions of this paper include:

(1) We propose a segmented ResNet based on the cloud, the edge, and the end devices, to try to solve the ReID problem at the local level;
(2) To enable the ResNet model to be deployed on the device side, we adopt the binarization approach, and to maintain the ReID’s accuracy, we add RSign and RPReLU on the basis of binarization to allow the network to automatically learn the distribution of the most appropriate binary thresholds and activation values using a learnable displacement.
(3) Through joint training, compared to the cloud-based ReID methods, the communication cost of our method is reduced, which is about 4–8×.

## 2. Related Work

In general, there are two categories of methods for ReID: a feature extraction method used to describe pedestrian images in the gallery library and query library and the distance metric method for comparing features among different images. ReID’s methods generally focus on two aspects to improve the recognition accuracy: one is to find a good distinguishable feature, and the other is to improve the similarity metric for comparing features, or to improve both aspects.

There are many research works [13,17] on representation learning. This is mainly due to the rapid development of convolutional neural networks (CNN), which can automatically extract the representative features required by the task from the original images. Therefore, some researchers regard ReID as a classification and verification problem. Geng et al. [18] used the classification network to predict the ID of the images and calculated the classification loss according to the predicted ID, then by verifying the characteristics of the two images combined by the network, and it is judged whether the two images belong to the same pedestrian. However, Lin et al. [13] believed that pedestrian ID information is not enough to learn a model with strong generalization ability. In addition, they added some attributes of the pedestrian, such as dress, hair style, and so on. In this way, the model not only has to predict the ID of the pedestrian, but also has to predict the attributes of the pedestrian.

In addition, the method of metric learning is also very popular. As a method of image retrieval, metric learning is different from representation learning, and its purpose is to learn the similarity of two images through the network. In the loss function of the network, the distance between the images of the same pedestrians (positive sample pairs) is as small as possible, and vice versa. The typical methods of metric learning loss are contrast loss [19], triple loss [14,20,21], quaternion loss [22], difficult sample sampling triple loss [23], and boundary mining loss [24].

The above work on ReID is based on global features, but some scholars have begun to explore approaches based on local features. For example, Varior et al. [25] cut the image and extracted local features, and Zhao et al. [26] used skeleton key points to locate, and so on. In addition, Wang et al. [27] used multiple frames of images to extract spatial features for training. What is more, the recent emerging method based on GAN [28] uses the data generated by itself to solve some difficulties in ReID.

**Figure 1:**
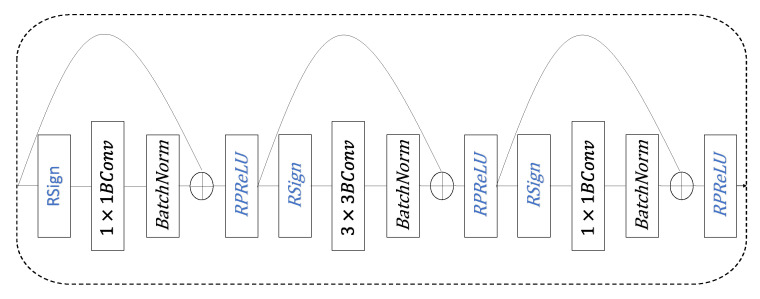
The block of ResNet, which combines RSign and RPReLU. BConv, binarized convolution.

All the methods mentioned above are carried out in an ideal environment in the cloud, but the task of ReID is bound to face practical application. In the actual situation, all kinds of resources are limited. If the neural network model for ReID is deployed in the cloud, the input pedestrian images will be transmitted from remote end devices to the cloud, which will bring a greater communication cost, while the fluctuation of the wide area network may bring a greater delay cost. If the neural network model is deployed on the end devices, the whole inference will hardly be carried out. There are two reasons: (1) End devices are limited in energy consumption, while the energy consumption of computation-intensive tasks is generally large; (2) end devices’ limited computing power may not be able to meet the user’s requirements for delay. Even though ResNet can be deployed on some end devices with sufficient resources, it will produce high costs and have no great practical significance. In response to limited end devices, we propose a segmented ResNet neural network model based on the three ends to implement ReID tasks. We sink the ResNet originally deployed on the cloud to the edge and the end devices by dividing the whole ResNet model into three parts and mapping it to the corresponding parts. In order to solve the problem of the lack of computing and other resources that may exist on the end devices, we binarize ResNet. In other words, ResNet based on the binary neural network (BNN) can reduce the computational density in the inference and be deployed on the end devices. BNN refers to the neural network obtained by binarizing the weight values and all the activation function values in its weight matrix (the value is limited to zero or one) on the basis of a floating-point neural network. In this paper, we adopt the binarization method of ReActNet [29]. BNN can binarize the weight matrix so that each weight takes up only one bit. Compared with the single-precision floating-point weight matrix, the memory consumption of the network model can be 32 times less in theory. Although the memory consumption of BNN (such as XNOR-Net [30]) is greatly reduced, it is difficult to carry enough characteristic information and ensure that the output range is suitable for the next level of binarization at the same time for BNN convolution output feature maps. In other words, if you directly sign the real values transferred in the network, you may encounter a low amount of information carried by the binary feature map due to the inappropriate range of real values. In this article, we combine RSign and RPReLU (shown in Figure 1) from ReActNet [29] to allow the network to automatically learn the distribution of the most appropriate binary thresholds and activation values using a learnable displacement. The accuracy of this network can be improved by such means.

**Figure 2:**
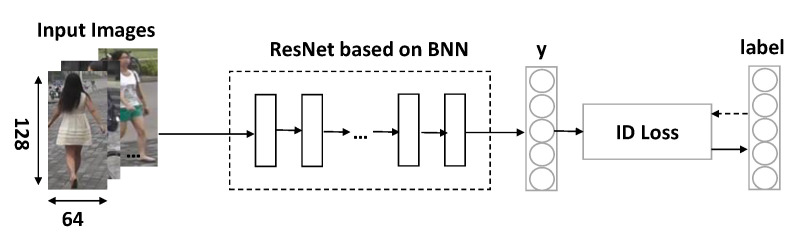
The frame diagram of ReID is realized based on classification. Take the pedestrian image (from dataset Market-1501) as the input; after binary ResNet50 layer network processing, use softmax as the classifier to get a prediction vector y; next, combine with the actual tag label to get the loss for back propagation; finally, get the trained target neural network. BNN, binary neural network.

Some BNN studies [30,31,32] have shown that on some datasets (such as the MINST dataset), the accuracy of using BNN is similar to that of the floating-point neural network, but the memory and computing resources are much smaller than the latter. We use binarized ResNet, in order to solve the problem of the lack of computing resources on the end devices. For the ReID method, we use representation learning to deal with the ReID problem as a classification problem, as shown in Figure 2.

The remainder of this paper is organized as follows. We first introduce the development of ReID and discuss the motivation of our method in Section 2. The main work of our segmented ResNet method, training and inference, is presented in Section 3. The experience compared to some traditional ReID methods and results are discussed in Section 4. Finally, we conclude this paper in Section 5.

## 3. A Binarized Segmented ResNet Based on Edge Computing for Re-Identification

In this part, we systematically introduce our proposed ReID method and the process of training and inference.

### 3.1. Segmented ResNet

**Figure 3:**
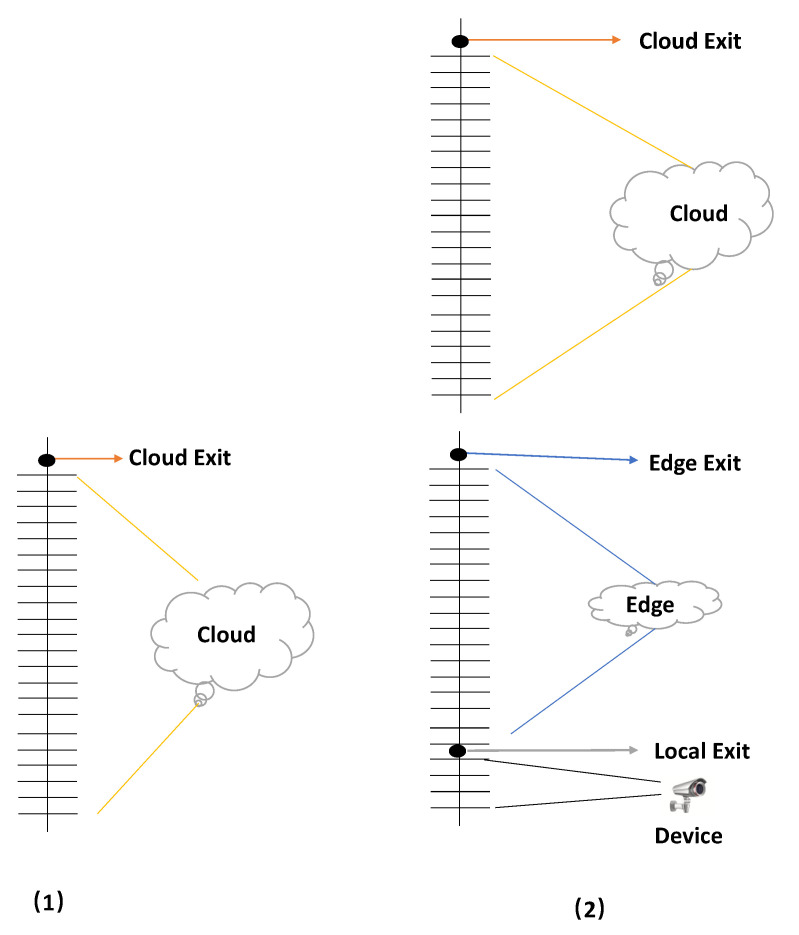
Conceptual model of the segmented neural network. Among them, Figure (**1**) is the cloud-based model, and Figure (**2**) is our improved layered model based on end devices, the edge and the cloud.

In order to solve the ReID problem, we adopt the ResNet model. The method we propose is to segment and map ResNet to the corresponding cloud, edge, and end devices. In addition, we refer to the network structure of BranchyNet [33] and add the exit points. The exit point exists on the physical devices, as shown in Figure 3. It is the place where pedestrian samples exit. If the sample processed by the local sub-network can meet the threshold requirements, it will exit at the local. Otherwise, it is necessary to continue to upload the intermediate results to the high-level network (the edge sub-network). The input sample will eventually exit at a certain exit point, ending this inference. As shown in Figure 3(1), the mainstream ReID methods are to deploy the entire neural network model on the cloud for inference. These methods can achieve higher ReID accuracy, but transferring the pedestrian image data collected on the end devices to the cloud for inference and returning the results will cause high data transmission cost and high delay caused by network fluctuations. On this basis, we improved the network. As shown in Figure 3(2), in addition to the cloud, the end devices are also added. The neural network (ResNet in this paper) is divided into two parts, which are mapped to the edge and the end devices, respectively. Firstly, the pedestrian images collected by the devices will be processed in the local network, and we will judge whether they reach the predetermined threshold. If so, they will exit at the local exit point, so that it is not necessary to upload intermediate results (feature maps) to the edge for processing, which greatly saves communication costs. If the predetermined threshold is not met, the intermediate results will be transmitted to the edge, and the deeper network (the cloud sub-network) will be used for inference, while input samples will exit at the edge exit point. Based on the end devices and the edge, the segmented ResNet can greatly improve the communication cost and the real-time performance. However, ReID’s recognition accuracy may not be ideal. Therefore, combined with the cloud, where a complete ResNet can be deployed, some samples that do not meet the threshold of the edge can be transmitted to the cloud for inference. However, the inference over the cloud will lead to the large difference in delay, as well as the large communication data overhead. The main focus of this paper is to solve the ReID problem on the edge, so the cloud inference part is not considered, but only to provide a solution for the samples that are difficult to identify by the collaborative inference of the edge and end devices.

### 3.2. Training

In order for the inference to be completed in the next section, we must train the segmented ResNet first. After training, ResNet can be deployed on the corresponding cloud, edge, and end devices for inference. The training was conducted on a server, which can ensure the efficiency of training. Since the segmented ResNet we propose has a part of the network on each side, there is a sub-loss at each corresponding exit point. Therefore, the training of the model requires joint training of the sub-loss on each part network, and finally, a network with high recognition accuracy is obtained. Our model is trained on a cloud server (Tesla P100). During the training, the sub-losses of all exits are combined for back-propagation so that the whole network can be jointly trained and each sub-network can also achieve great accuracy. More details about the joint training can be found for BranchyNet [33] and GoogLeNet [11]. We trained ReID as a multi-classification task and used cross-entropy as the loss function, as follows:

(1) $$L\left(y,y^{\prime };\theta \right)=-\frac{1}{N}\sum_{i=1}^{N}y_{i}\log y_{i}^{\prime }$$

where *N* represents the number of categories of pedestrians during training and θ represents the network parameters such as the weights and biases of the layers of the network; *y* represents a one-hot ground-truth label vector, and $y^{\prime }$ is a one-hot probability vector. However, it is finally generated by each input through each exit point during the training process and can be obtained by calculating the value of the softmax function, as follows:

(2) $$y_{i}^{\prime }=\frac{e^{z_{i}}}{\sum_{j=1}^{N}e^{z_{j}}}$$

where $y_{i}^{\prime }$ is the probability of the *i*-th category in the $y^{\prime }$ predicted probability vector, *z* represents the result from the entrance of the entire network to an exit point, and $z_{i}$ represents the value of the *i*-th value of *z*. The entire function $f_{n}$ is a function representing the computation of the neural network layers from an entry point to the *n*-th exit point. The equation can be written as:

(3) $$z=f_{n}\left(x;\theta \right)$$

where *x* represents the input (pedestrian images) of the whole network. There are multiple exit points in our proposed network, which means that there are multiple sub-losses, and joint training is needed to jointly optimize the weighted sum of these sub-losses to minimize them. The combined loss is as follows:

(4) $$\begin{array}{cc} L(y,y^{\prime };\theta )= & \alpha_{l}L\left(y,y_{exit_{l}}^{\prime };\theta \right)+\alpha_{e}L\left(y,y_{exit_{e}}^{\prime };\theta \right) \end{array}$$

where $\alpha_{l}$ and $\alpha_{e}$ refer to the weight corresponding to the local exit point, respectively the edge exit point. Equal weights are used for the experimental section of this paper.

### 3.3. Inference

In the previous stage, we got ResNet, which has been trained. During inference, firstly, we will divide the trained ResNet into two parts according to the stochastic segmentation. We can conduct multiple experiments to determine the segmentation method of ResNet (i.e., Section 4.5). Then, we deploy two sub-networks with their parameters into the end devices and the edge, respectively. Next, a pedestrian image will be input into the segmented ResNet. The input image exits at the local or continues to transmit the intermediate results to the edge depending on a preset threshold *T*. The threshold of the local and the edge determine whether to transmit the intermediate results or exit. One way to define *T* is by searching over the ranges of *T* on a validation set and picking the one with the better accuracy and the better local exit rate. We use the following similarity function as the confidence criterion that determines whether a sample exits at a particular exit point. The similarity function is defined as:

(5) $$\rho \left(x\right)=\max_{1<i<N_{p}}\left(Sim\left(a,b_{i}\right)\right)$$

(6) $$Sim\left(a,b_{i}\right)=\frac{a^{T}b_{i}}{\sqrt{a^{T}a}\sqrt{b_{i}^{T}b_{i}}}$$

where $N_{p}$ represents the number of pedestrian images collected on the local server that have been collected all the time and $Sim(a,b_{i})$ represents the similarity between the given input pedestrian image and a pedestrian in the server library of pedestrian images during inference. *a* is the feature vector generated by the local network processing of the input pedestrian image during inference, and $b_{i}$ represents the feature vector generated by a pedestrian passing through the local network in the local server library of pedestrian images. At the exit point, the feature vector generated by the input sample will be calculated using Equation (6). The calculation result ρ(x) will be compared with the threshold *T*. If the requirement of *T* is met (i.e., ρ(x)>T), the input sample can exit at this exit point. The value of ρ(x) ranges from zero to one. If it is closer to one, the more confident the segmented ResNet is in finding the matching pedestrian, and vice versa. At each exit point, there is a threshold *T*, which will determine whether the input pedestrian image exits at the exit point. This judgment will be made at each exit point to determine whether to continue to transmit the intermediate results to a deeper network, or it can be confident to exit at this segment of the network (i.e., ρ(x) > *T*). For some cases requiring high precision, input samples that are not satisfied on the edge can be transmitted to the cloud for inference with full ResNet and exit at the cloud exit point. In any case, the input sample will eventually exit at the last exit point to end the inference.

Algorithm 1 above is the situation of inference in the presence of multiple devices under the cloud, the edge, and the end devices.
**Algorithm 1:** A binarized segmented ResNet based on edge computing for ReID.
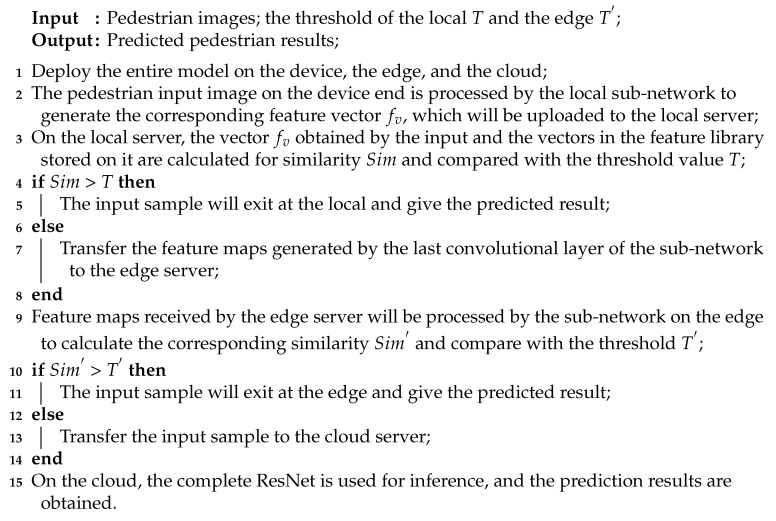


**Figure 4:**
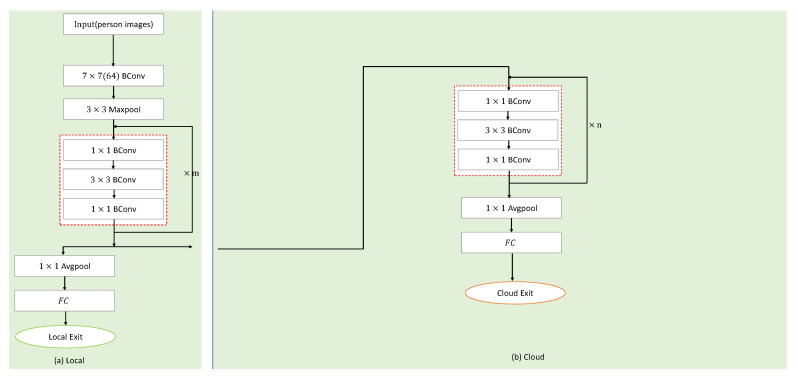
The segmented ResNet50 inference framework based on the end devices and the edge. The input is a pedestrian image. The left part (**a**) is the inference part on the end devices, and the right side (**b**) is the inference part on the edge.

The inference framework used in the experiment in this paper is as shown in Figure 4. In the experiment, we demonstrate the feasibility of splitting ResNet on the device and the edge.

In the latter section of the experiment, we also use the local and the edge segmentation framework to solve the ReID problem. What is more, ResNet adopts a 50 layer structure, where the input is a pedestrian image, and the BConv block is a binarized convolution operation, while Maxpool is the maximum pooling, Avgpool the average pooling, and FC the fully connected layer. Whether to exit at the local depends on the threshold *T*. The red dotted box represents the basic residual block of ResNet. There are four types. The ×m and ×n above it represent the number (*m*,*n*) of residual block iterations.

### 3.4. Inference Communication Cost

In the inference process, the communication cost from the device to the local server and the edge can be expressed by the following equation:

(7) $$c=\frac{\left|V\right|}{8}+\left(1-\eta \right)\frac{f\times o}{8}$$

where *V* represents the feature vector generated by the input pedestrian image passing through the local network, η is the exit rate of the input pedestrian image at the local, and *f* represents the number of filters of the convolutional layer that produce intermediate results (feature maps). *o* represents the size of the output produced by a single filter. The denominator 8 corresponds to the bits used to express a byte output. As shown in Equation (7), the cost is divided into two parts. The first term is the communication cost caused by the transmission of pedestrian image features from the devices to the local server. This part is carried out regardless of whether the input sample is confident enough to exit locally. The second term is the communication between the local server and the edge, which happens a (1−η) fraction of the time, when the input sample exits at the edge rather than the local. This cost term is closely related to the local exit rate.

## 4. Experimental Evaluation

In this section, we verify the performance of our binarized segmented ResNet in recognition accuracy and communication data transmission in the ReID scenario.

### 4.1. The Segmented ResNet50 Based on Multiple Devices in the ReID Scenario

**Figure 5:**
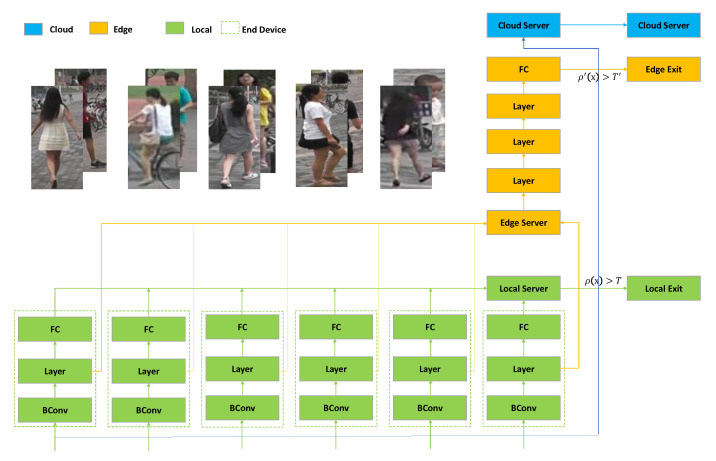
A segmented ResNet50 layer network framework for inference under the ReID scenario. And the images in this figure are from Market-1501.

Figure 5 shows the overall framework of our network (we take an example of a 10 layer local sub-network). The input of the whole model is the pedestrian image collected from the end devices. In the figure, the green, the orange, and the blue blocks represent parts of the network structure that run on the end devices, the edge, and the cloud. In the figure, BConv represents the convolutional layer, Layer represents a large convolutional layer in the ResNet50 network, and FC represents the fully connected layer. The green dotted box represents an end device. In addition, there are six end devices, and the pedestrian pictures collected by these six devices are transmitted to the local server, which forms the pedestrian library to be identified. The Layer layer contains several convolutional layers, and the number of convolutional layers varies from layer-to layer. However, they are all formed by several residual blocks. Inside the block are three convolutional layers, namely the convolutional layers whose convolution kernel sizes are 1×1, 3×3, and 1×1. The Layer at the end devices (i.e., the green block) is composed of three residual blocks, all of which have nine convolutional layers. The three Layer blocks from bottom to top of the edge are composed of 4, 6, and 3 blocks, respectively, namely 12, 18, and 9 convolutional layers. In this paper, an additional local server is used, not as an edge computing platform, but simply to store the processed pedestrian image data on the associated local devices for subsequent inference and to determine whether the samples exit in the local. In the local server, the feature vectors transmitted by each device will be found from the pedestrian library for similarity calculation. If the final similarity is greater than the given threshold *T* (ρ(x)>T), this indicates that the local end devices are relatively confident in the recognition result, and the sample can exit at the local exit point. Otherwise, the results generated by the last convolutional layer will be transmitted to the edge for inference.

In Figure 5, although the end divides the 10 layers of the ResNet50 network, any general end device cannot meet the resource requirements of inference. Therefore, we performed a binary operation on the entire network. The convolutional layer BConv, as well as the standardization and activation functions omitted in the framework are also binarized. As a result, some network structures can be deployed on the local side. In this framework, there are multiple end devices, and their corresponding network structures are part of the local network in Figure 4.

In this experiment, due to the decrease of network recognition accuracy after binarization, we tried to choose the model with higher recognition accuracy for our comparative experiment. We considered the two models ResNet and Mobilenet [34], but after comparison, the unbinarized ResNet50 network can obtain ReID recognition accuracy of rank-1 as 87%, but the rank-1 of Mobilenet V2 is only 73.87%. Similar results were also verified by Marchwica et al. [35]. Perhaps the ResNet50 we chose is not the optimal network for ReID, but we were more concerned about the segmentation scheme and association reasoning applied in the network. What we present is a framework that actually works for most CNNs. In this article, we chose ResNet50 as the network model, of course MobileNetis also possible. We can minimize ResNet’s computing and memory overhead on the device by binarization and deploying as few convolutional layers as possible on the device side. Therefore, we used the segmented ResNet50 network structure (i.e., the network in Figure 3 (2)) based on the end devices and the edge without loss of generality. ResNet can be flexibly divided into two parts based on the edge and end devices. If high recognition accuracy is required, we can add the cloud for the input samples, which cannot exit at the edge exit point to infer. In this paper, our research focus is not on accuracy, but on low communication cost and high real-time performance. Therefore, we set a high threshold of the exit point on the edge and let all samples exit on the edge or end devices. If high precision is required for some scenarios, the threshold on the edge can be adjusted appropriately.

During joint training, some hyperparameters were configured as follows. The batch size was set to 32, and the learning rate was set to 0.05. The epochs were set to 250. Besides, because we used the split ResNet based on the end devices and the edge in the experiment, the values of $\alpha_{l}$ and $\alpha_{e}$ were set to 0.5.

### 4.2. Hardware Selection

**Figure 6:**
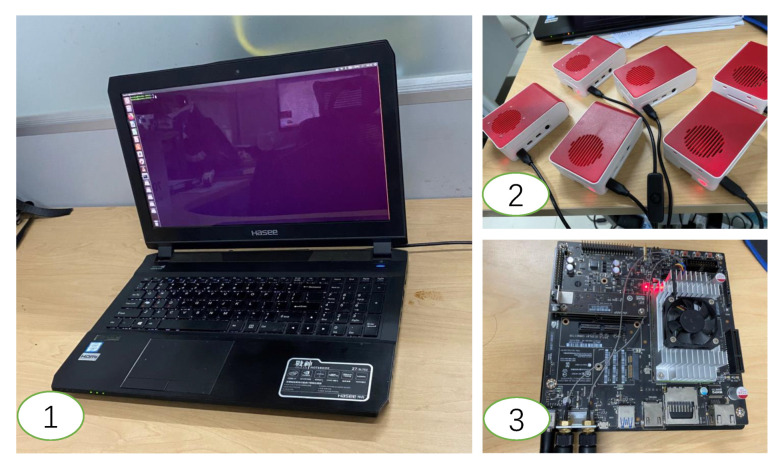
The three categories of hardware. Subfigures (**1**–**3**) show the Raspberry 4b, Jetson TX2, and the laptop, respectively.

In order to complete our experiment, we adopted three types of hardware in total, shown in Figure 6, including six Raspberry Pi 4B, one Jetson TX2, and one laptop. In addition, we used a cloud server with two Tesla P100 GPUs.

The six Raspberry Pi 4B were used as the six end devices to collect pedestrian images, which were obtained from the six cameras of Market-1501. At the same time, the local sub-network was deployed on the six Raspberry Pi 4B to complete the acquisition of the feature maps. The Jetson TX2 was used as the local server to store the pedestrian feature maps processed by the local sub-network from the six Raspberry Pi 4B, and to judge whether the input sample can exit at the local or not. The laptop was used as the edge node, which was deployed in the edge sub-network. What is more, the server with Tesla P100 was used as the cloud, where we trained the entire ResNet50 and decided whether to use it for inference according to the accuracy requirements of the application. We describe the detailed configuration information of these devices in Table 1.

### 4.3. Dataset

We conducted experiments on Market-1501 [36] and DukeMTMC-ReID [28]. The dataset Market-1501 includes a rectangle frame of 1501 pedestrians and 32,668 detected pedestrians captured by 6 cameras (5 high-definition cameras and 1 low-definition camera). Each pedestrian is captured by at least 2 cameras, and there may be multiple images in one camera. The training set has 751 people, containing 12,936 images, and each person has an average of 17.2 training data; the test set has 750 people, containing 19,732 images, and each person has an average of 26.3 test data. The input of training and inference was 128 × 64 RGB pedestrian images.

The dataset DukeMTMC-ReID is a subset of the newly-released multi-target, multi-camera pedestrian tracking dataset. The original dataset contains eight 85 minute high-resolution videos from eight different cameras. Hand-drawn pedestrian bounding boxes are available. The DukeMTMC-ReID dataset for ReID has 1812 identities from eight cameras. There are 1404 identities appearing in more than two cameras and 408 identities (distractor ID) that appear in only one camera. It randomly selects 702 IDs as the training set and the remaining 702 IDs as the testing set. In the testing set, it picks one query image for each ID in each camera and puts the remaining images in the gallery.

### 4.4. Selection of Threshold T

There is a threshold *T* at each exit point, except the exit point on the cloud. The inference will be completed anyway. The exit point represents the confidence level that the input sample can achieve the expected goal and exit after processing in this segmented sub-network. In this experiment, we used similarity as the confidence criterion. Therefore, if the threshold value is 1, it is impossible for the similarity of any other pedestrian image pair to reach or exceed 1. In other words, no samples can exit at the exit point corresponding to this threshold *T*. On the contrary, if the threshold value is 0, then any input samples can meet the threshold condition and will exit at the corresponding exit points. Therefore at the local, no intermediate results are transmitted to the edge, to a certain extent reducing the communication overhead. However, in this case, the ReID accuracy will be very low because of the shallow network.

We need to choose an appropriate threshold *T* to achieve a better balance between ReID recognition accuracy and the communication cost of inference. The cost of communication is given in Equation (7), and the term of the cost caused by the transmission of pedestrian image features from the end devices to the local server is inevitable. We only reduce the second term of the samples that the local side failed to exit. The intermediate result of continuing to be transmitted to the edge is the communication cost. This requires us to improve the local exit rate as much as possible.

**Figure 7:**
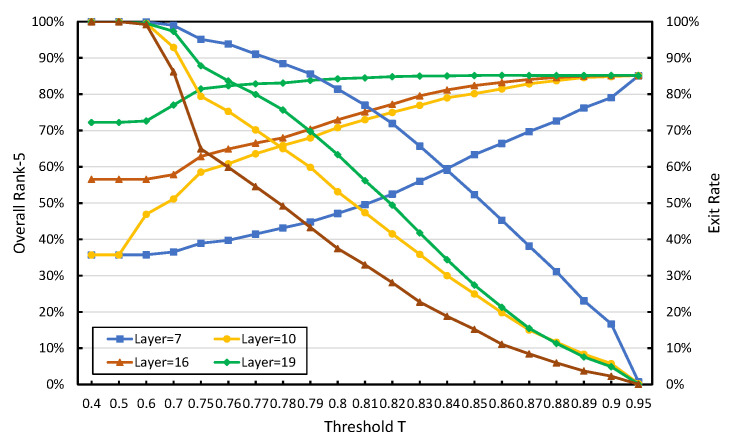
Line chart based on threshold *T* and local exit rate under 4 different segmentation methods.

Figure 7 shows the relationship between the value of threshold *T* and the local exit rate η of the sample and *T* and the overall rank-5 accuracy under 4 kinds of ResNet50 network segmentation methods. The *L* in the figure represents the number of convolutional layers in the local sub-network. As for the selection of the best segmentation point, we will introduce it in the next subsection.

There are 8 polylines in Figure 7. Their same two polylines represent the relationship between the value of threshold *T* and the local exit rate of the sample and *T* and the overall rank-5 accuracy under a ResNet50 network segmentation point. The former shows a downward trend, while the latter shows an upward trend. Under the 4 different segmentation schemes of ResNet50, the convolutional layers of the local (L) are 7, 10, 16, and 19, respectively. Although the binarization of the entire ResNet reduces the resource demand pressure of the neural network deployed on the local end devices, the resource availability of the local sub-network is still limited. In the experiment, we present 4 kinds of segmented methods, and the end devices can meet the required resources. In a real-world scenario, you can make adjustments based on the actual situation.

As shown in Figure 7, when the local network has only 7 layers and the threshold *T* is less than 0.5, basically all input pedestrian samples will exit at the local exit point. However, due to the local shallow network, the recognition accuracy is low (rank-5 is only about 35.7%). When *T* is greater than 0.95, basically no input pedestrian samples can exit at the local exit point. All input samples will generate intermediate results through the local 7 layer network, and the intermediate results will be transmitted to the edge. The remaining 43 layers of the network will be used for further inference at the edge. In this case, the recognition accuracy is very high, and rank-5 is around 70.8%; however, the communication data will be very expensive (see Table 2). The standard for selecting the *T* value is to strive for good identification accuracy while trying to make pedestrian samples exit at the local (with a high exit rate). As shown in the figure, as the value of *T* gets larger and larger, the local exit rate will also get smaller and smaller, and the overall identification accuracy will also get higher and higher. Combining the corresponding cost Table 2, the *T* value is set to 0.85. At this time, the local exit rate is 52.32%, and the total rank-5 is 63.33%. In the case of a shallow local network, the local exit rate has to be increased in order to obtain higher identification accuracy. Although the overall rank-5 is much smaller than the 95.49% accuracy achieved with the full network, the communication overhead is only 7845 bytes, reducing the communication cost by a factor of 4×.

In Figure 7, the overall trend of the local exit rate and overall rank-5 is consistent when the threshold *T* is evaluated from 0 to 1. However, when *T* is less than 0.5, the actual accuracy of the overall rank-5 is quite different. This is determined by the depth of the neural network model deployed on the local. In other words, the local network goes from Layer 7 to Layer 10, Layer 16, and Layer 19, and the local rank-5 keeps growing. When L is 10, 16, and 19, the threshold *T* is set to 0.79, 0.8, and 0.75, respectively, after the comprehensive equalization of the communication cost and ReID accuracy combined with Table 3, Table 4 and Table 5, respectively. The final accuracy of rank-5 is 60.5%, 69.34%, and 85.23%, respectively, and the corresponding communication cost is 6620 bytes, 3044 bytes and 2914 bytes.

### 4.5. Selection of the Segmentation Point

**Figure 8:**
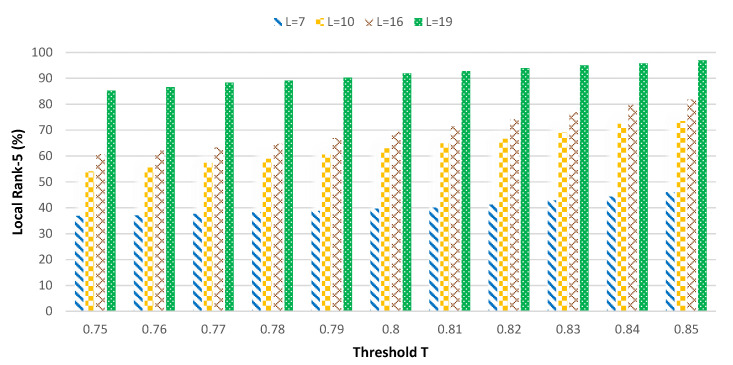
Histogram of the local rank-5 based on four segmentation points.

In the previous subsection, we analyzed the effects of 4 different segmentation methods on the local sample exit rate and overall rank-5 accuracy. In Figure 8, we compare the recognition accuracy (rank-5) of local samples at 4 different segmentation points with the local sample exit rate.

Figure 8 is the histogram of the local rank-5 and segmentation point. The 4 different types of histograms represent the 4 different segmentation methods. We show that with the threshold *T* ranging from 0.75 to 0.85 for any selected value, the local rank-5 keeps growing with the deepening of the local network (from 7, 10, 16, to 19 layers). The depth of the local network is very important for the improvement of the local network identification accuracy.

**Figure 9:**
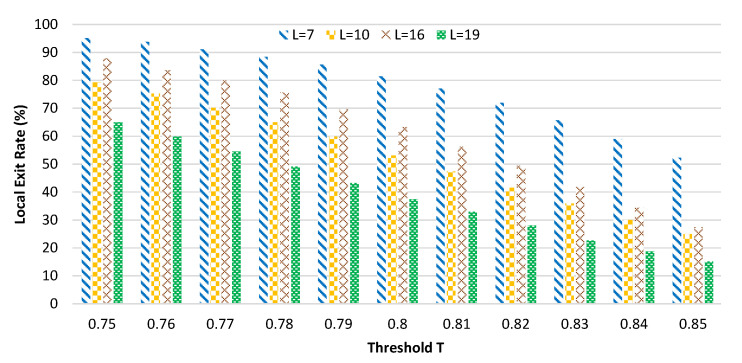
Histogram of the local based on four segmentation points.

Figure 9 is the histogram of the local sample exit rate and segmentation point. The 4 different types of histograms represent the 4 different segmentation methods. The experimental results are similar to those in Figure 8. The threshold value is between 0.75 and 0.85. With the deepening of the local network, the exit rate of the local sample also increases.

Therefore, in the next experiment, we chose the 19 layer network for the local network, which maximized the identification accuracy and the local sample exit rate (which indirectly reduced the communication cost). However, in the actual scenario, the network should be selected based on the available resources of end devices to deepen the local network as much as possible.

### 4.6. Experimental Results on the Dataset DukeMTMC-ReID

In the first few sections of the experimental section, we introduced how our model is segmented and the selection of threshold *T* using the dataset Market-1501. Finally, we chose the 19 layer network for the local network and set *T* to 0.75.

Then, we repeated the above experiment with DukeMTMC-ReID as the dataset. We found that as the network layer of the local network deepens, higher recognition accuracy can be achieved. Therefore, we also choose the 19 layer network for the local network. For the threshold *T*’s setting, refer to Table 6. This table shows the effect of different T values on the local, overall rank-5, and communication data cost where the local sub-network choice is the 19 layer network. With the increase of the T value, the local exit rate of the sample decreases, and the overall rank-5 and communication cost increase. In order to balance the communication cost and recognition accuracy, we tried our best to exit the input pedestrian sample at the local. Finally, we set *T* to 0.83.

### 4.7. Comparison with the Baseline

From Table 7, we can see that the recognition accuracy (rank-5) of our proposed method on Market-1501 is 81.47%, and those of rank-1, rank-10, and mean Average Precison (mAP) are 59.95%, 87.35%, and 41.37%, respectively. Baseline 1 is the result of the original ResNet50 on the cloud, while Baseline 2 is the result of ResNet50 with binarization on the cloud. It can be seen from Baseline 1 and Baseline 2 that, after binarization, the complete ResNet50 has a reduction of about 20 percentage points in rank-1, but only a reduction of about 10 percentage points in rank-5. In order to mitigate the reduction in accuracy caused by adopting binarization, we used rank-5 as ReID’s precision measurement standard. In our approach, we used the segmented ResNet50 binarization to resolve ReID. Regarding the segmentation methods, we chose the 19 layer network for the local and set the exit threshold at the local exit point as 0.75. The final results are shown in Table 7. Since most of the input pedestrian samples of our model will exit at the local and the local network is only part of ResNet50, the overall accuracy will be slightly lower than the results of the cloud-based model. It was about six percentage point lower at rank-1. However, our method achieved good results in terms of communication cost. Compared with the deployment of a complete neural network model on the cloud, the proposed method can reduce the transmission overhead of uploading pedestrian image data on the device to the cloud. A 128×64 RGB pedestrian image was input and transmitted to the cloud for inference. Sending a 128×64 RGB pixel image (the input size of our dataset) to the cloud cost 24,576 bytes per image sample. As shown in Table 5, when the exit point threshold *T* was set to 0.75 under the condition of equating the accuracy of rank-5 and communication cost, the communication transmission cost in this experiment was 2914 bytes, which reduced the communication cost by a factor of 8×.

In addition, we did a comparison experiment on MobileNet. There are four segmentation schemes on MobileNet, and the corresponding numbers of the local network layers are: 5, 9, 13, and 17. Similarly, we adopted the 13 layer local network segmentation method and set the threshold of the local as 0.86. The results can be seen in Table 7. As you can see from the table, with the application of MobileNet to our method, due to the fact that the original MobileNet is not very high in precision, the precision of MobileNet deployed on the end devices and the edge after binarization and segmentation is much lower than our ResNet50-based method. However, the MobileNet-based method has also achieved good results in terms of communication cost. In addition to the communication overhead, our approach also improved significantly in terms of computation. On ReID, the traditional ResNet50 model requires 2.69 GFLOPS computation, while our ResNet50-based method only requires 0.25 GFLOPS, and the MobileNet-based method only requires 0.31 GFLOPS. In other words, we improved the computational performance by about a factor of 10. This provides great convenience for deploying resource-limited models on the edge and end devices.

During the inference, we took 3368 pedestrian images as the input samples of the whole network, among which, 2187 samples exited at the local server, while the remaining 1181 samples exited at the edge. During the inference, the computation amount of the local sub-network was 0.11 GFLOPS, while the computation amount of the edge sub-network was 0.14 GFLOPS.

As for Table 8, we used DukeMTMC-ReID to replace Market-1501, and everything else was the same. In addition, we chose the 19 layer network for the local network and set *T* to 0.83. As DukeMTMC-ReID is more complex than Market-1501, its recognition accuracy was reduced. The original network after binarization brought more precision loss. When we substituted MobileNet with ResNet50, we adopted the 13 layer local network segmentation method and set the threshold of the local as 0.79. However, the MobileNet-based method was not as accurate as the ResNet-based method. Using our ResNet-based method, although the accuracy of rank-5 was much lower than that of Baseline 1, it had 5 times the improvement in the communication cost, and the calculation amount dropped from 2.69 GFLOP to 0.25 GFLOP.

## 5. Conclusions

In this paper, we propose a binarized segmented ResNet50 network based on three ends to solve the ReID problem in actual scenarios. Our method is suitable for ResNet with the deep network layers of the local sub-network. In the previous work, most of the ReID methods improved on the algorithm and strove to improve the recognition accuracy (such as rank-n, mAP) of ReID in an ideal cloud environment. Our method is to segment the ResNet50 network and map it to the corresponding three ends. Then, we use the trained model obtained by joint training to conduct inference. The threshold *T* at the exit point on each end is set to determine whether the input pedestrian data on each end device exit in advance at the corresponding exit point. To reduce the communication cost of the entire inference, we try to exit at the local.

Finally, the experimental results can prove that our proposed method can reduce the overhead of transmitting communication cost by 4–8× while ensuring that the recognition accuracy of ReID is not much lower than the current better ReID methods. Besides, the binary ResNet adopted by us processes most of the data close to the devices producing the data and exit, which reduces the possibility of privacy leakage to a certain extent. In ReID real scenarios with high communication requirements, our method will have better performance. 

## Figures and Tables

**Table 1:** Hardware setup and configuration.

	Raspberry Pi 4B	Jetson TX2	Laptop	Server
CPU	Broadcom BCM2711B0	ARMv8	Inter Core i7-6700HQ	Inter Xeon E5-2630 v4
GPU	VideCore VI	NVIDIA Pascal	NVIDIA GTX970M	Tesla P100
Frequency	1.5 GHz	2 GHz	2.6 GHz	2.2 GHz
Core	4	6	4	10
Memory	4 GB	8 GB	16 GB	62 GB
OS	Raspbian	Ubuntu16.04	Ubuntu16.04	Ubuntu16.04

**Table 2:** While local network L = 7, the effects of different exit thresholds (*T*) settings for the local. *T* = 0.85 is used in L = 7.

T	Local Exit (%)	Rank-5 (%)	Comm. (B)
0.4	100	35.72	64
0.5	100	35.72	64
0.6	99.94	35.74	74
0.7	98.96	36.03	234
0.75	95.16	36.8	854
0.76	93.82	37.06	1072
0.77	91.09	37.65	1518
0.78	88.45	38.27	1949
0.79	85.63	38.8	2409
0.8	81.44	39.63	3093
0.81	76.99	40.07	3093
0.82	71.94	41.23	4643
0.83	65.71	42.84	5660
0.84	59.06	44.24	6745
**0.85**	**52.32**	**46.08**	**7845**
0.86	45.25	46.92	8999
0.87	38.15	48.33	10,158
0.88	31.09	48.81	11,310
0.89	23.1	50	12,614
0.9	16.69	52.31	13,660
0.95	0.71	70.83	16,268

**Table 3:** While local network L = 10, the effects of different exit thresholds (*T*) settings for the local. *T* = 0.79 is used in L = 10.

*T*	Local Exit (%)	Rank-5 (%)	Comm. (B)
0.4	100	46.64	64
0.5	100	46.64	64
0.6	99.55	46.85	137
0.7	92.90	49.63	1222
0.75	79.42	54.06	3422
0.76	75.27	55.5	4100
0.77	70.19	57.4	4929
0.78	65.02	58.9	5772
**0.79**	**59.83**	**60.5**	**6620**
0.8	53.12	62.94	7715
0.81	47.33	64.87	8660
0.82	41.51	66.6	9610
0.83	35.87	68.96	10,531
0.84	29.99	72.38	11,490
0.85	24.94	73.45	12,314
0.86	19.77	74.77	13,157
0.87	15.02	79.05	13,932
0.88	11.64	81.89	14,485
0.89	8.34	88.26	15,022
0.9	5.73	90.16	15,449
0.95	0.21	100	16,350

**Table 4:** While local network L = 16, the effects of different exit thresholds (*T*) settings for the local. *T* = 0.8 is used in L = 16.

*T*	Local Exit (%)	Rank-5 (%)	Comm. (B)
0.4	100	56.56	64
0.5	100	56.56	64
0.6	99.55	56.56	100
0.7	97.36	57.52	279
0.75	87.86	60.7	1051
0.76	83.67	62.03	1391
0.77	79.96	64.61	2040
0.78	75.68	58.9	5772
0.79	69.74	66.92	2523
**0.8**	**63.33**	**69.34**	**3044**
0.81	56.18	71.47	3626
0.82	49.41	74.16	4176
0.83	41.78	76.97	4796
0.84	34.38	79.79	5397
0.85	27.4	81.91	5965
0.86	21.29	84.38	6462
0.87	15.47	88.29	6935
0.88	11.31	92.13	7273
0.89	7.57	95.29	7577
0.9	4.9	97.58	7794
0.95	0.09	100	8185

**Table 5:** While local network L = 19, the effects of different exit thresholds (*T*) settings for the local. *T* = 0.75 is used in L = 19.

*T*	Local Exit (%)	Rank-5 (%)	Comm. (B)
0.4	100	72.24	64
0.5	100	72.24	64
0.6	99.23	72.27	127
0.7	86.19	78.44	1186
**0.75**	**64.93**	**85.23**	**2914**
0.76	59.83	86.61	3329
0.77	54.54	88.35	3759
0.78	49.17	89.13	4196
0.79	43.29	90.33	4673
0.8	37.47	91.92	5146
0.81	32.96	92.79	5513
0.82	28.03	93.96	5914
0.83	22.71	95.03	6346
0.84	18,76	95.73	6667
0.85	15.17	96.87	6959
0.86	11.07	97.86	7292
0.87	8.4	97.88	7509
0.88	5.94	97.5	7709
0.89	3.68	96.77	7893
0.9	2.29	97.4	8006
0.95	0.03	100	8190

**Table 6:** While local network L = 19, the effects of different exit threshold (*T*) settings for the local. *T* = 0.83 is used in L = 19 (DukeMTMC-ReID).

*T*	Local Exit (%)	Rank-5 (%)	Comm. (B)
0.4	100	37.61	64
0.5	100	37.61	64
0.6	99.64	37.61	93
0.7	97.31	38.19	284
0.75	91.65	40.35	748
0.76	89.72	41.2	906
0.77	87.66	42.06	1075
0.78	83.93	43.4	1380
0.79	80.3	44.7	1678
0.8	75.13	46.77	2101
0.81	68.63	49.24	2634
0.82	61.45	51.08	3222
**0.83**	**51.89**	**53.95**	**4005**
0.84	44.08	55.75	4645
0.85	36.31	57.63	5281
0.86	29.22	59.47	5862
0.87	22.76	60.41	6392
0.88	17.64	61.36	6811
0.89	12.52	61.71	7230
0.9	8.44	62.07	7565

**Table 7:** Comparison with the baseline.

Method	Rank-1 (%)	Rank-5 (%)	Rank-10 (%)	mAP(%)	Comm. (Bytes)	FLOPS ($10^{9}$)
ResNet50 Baseline 1	88.24	95.49	97.06	72.28	24,756	2.69
ResNet50 Baseline 2	65.74	85.18	90.11	45.53	24,756	0.25
Our MobileNet	22.64	48.69	60.67	13.64	3396	0.31
**Our ResNet50**	**59.95**	**81.47**	**87.35**	**41.37**	**2914 (8×)**	**0.25**

**Table 8:** Comparison with the baseline using DukeMTMC-ReID.

Method	Rank-1 (%)	Rank-5 (%)	Rank-10 (%)	mAP (%)	Comm. (Bytes)	FLOPS ($10^{9}$)
ResNet50 Baseline1	77.6	88.69	92.06	60.3	24,756	2.69
ResNet50 Baseline2	43.4	62.39	70.42	27.05	24,756	0.25
Our MobileNet	19.0	35.66	45.11	11.73	3708	0.31
**Our ResNet50**	**32.41**	**53.95**	**63.56**	**23.58**	**4005 (5×)**	**0.25**
